# A Survey of System Architecture Requirements for Health Care-Based Wireless Sensor Networks

**DOI:** 10.3390/s110504875

**Published:** 2011-05-03

**Authors:** Emeka E. Egbogah, Abraham O. Fapojuwo

**Affiliations:** Department of Electrical and Computer Engineering, University of Calgary, 2500 University Drive NW, Calgary, AB T2N 1N4, Canada; E-Mail: fapojuwo@ucalgary.ca

**Keywords:** wireless sensor networks, healthcare, quality of service (QoS), MAC protocols, routing protocols, end to end reliable transport protocols, network coding

## Abstract

Wireless Sensor Networks (WSNs) have emerged as a viable technology for a vast number of applications, including health care applications. To best support these health care applications, WSN technology can be adopted for the design of practical Health Care WSNs (HCWSNs) that support the key system architecture requirements of reliable communication, node mobility support, multicast technology, energy efficiency, and the timely delivery of data. Work in the literature mostly focuses on the physical design of the HCWSNs (e.g., wearable sensors, *in vivo* embedded sensors, *et cetera*). However, work towards enhancing the communication layers (*i.e*., routing, medium access control, *et cetera*) to improve HCWSN performance is largely lacking. In this paper, the information gleaned from an extensive literature survey is shared in an effort to fortify the knowledge base for the communication aspect of HCWSNs. We highlight the major currently existing prototype HCWSNs and also provide the details of their routing protocol characteristics. We also explore the current state of the art in medium access control (MAC) protocols for WSNs, for the purpose of seeking an energy efficient solution that is robust to mobility and delivers data in a timely fashion. Furthermore, we review a number of reliable transport layer protocols, including a network coding based protocol from the literature, that are potentially suitable for delivering end-to-end reliability of data transmitted in HCWSNs. We identify the advantages and disadvantages of the reviewed MAC, routing, and transport layer protocols as they pertain to the design and implementation of a HCWSN. The findings from this literature survey will serve as a useful foundation for designing a reliable HCWSN and also contribute to the development and evaluation of protocols for improving the performance of future HCWSNs. Open issues that required further investigations are highlighted.

## 1. Introduction

The budget for health care now accounts for a significant portion of several economies around the world. In the United States (U.S.) alone, $2.5 trillion dollars were dedicated to health care in 2009, amounting to approximately 17% of the gross domestic product (GDP) [1]. The 17% of GDP spent by the U.S on health care is almost double that of the GDP spent by much of the rest of the world (approximately 9%), even though the U.S utilizes fewer doctors and nurses per person [2]. Of each dollar spent on health care in the U.S., approximately 40% of it is consumed by hospital care and nursing homes [3]. There are several negative factors that contribute to the high costs of provisioning health care in hospitals and nursing homes, such as poor doctor and nurse to patient efficiency and the inability to constantly monitor a patient’s health. These factors not only contribute to rising health care costs, but may also play a role in health care related preventable deaths, which account for approximately 44,000 to 98,000 deaths per year in the U.S., as estimated by the Institute of Medicine (IOM) [4].

Technology has always presented ways of improving and enhancing our lives. Thanks to the continued realization of Moore’s law, innovation in the area of sensor technology has allowed the integration of tiny, low-power, and wearable smart medical sensor devices (e.g., pulse oximeters [5], electrocardiographs [6], and accelerometers [7]) into commonly used wireless sensor devices. These sensor-equipped wireless devices form wireless sensor networks (WSN) that today, offer a potential solution to the inefficiencies that plague the health care industry. In recent years, WSN-based health care systems have been deployed for applications such as home monitoring for chronic and elderly patients [8], real-time continuous patient monitoring in hospitals [9], automated vital sign analysis to reduce the incidents of medical accidents due to human error [10], and emergency situations [11]. For most applications requiring the design, implementation, and deployment of WSNs, the following challenges inherent to almost all WSN applications must be overcome: low computational power, poor communication bandwidth, congested wireless medium, and limited energy budget. However, health care related applications for nursing homes, emergency scenarios, or hospitals require more specific requirements to make the integration of WSNs successful.

There are five main requirements that the architecture for a health care WSN (HCWSN) must satisfy: (1) reliability—The ability to transmit accurate and diverse data while meeting stringent quality of service (QoS) requirements, in terms of high packet delivery ratio (PDR) and low end-to-end latency, is of paramount importance in medical settings. (2) Energy Efficiency—One key draw of WSNs to health care applications is that many sensors do not require external power (*i.e*., they use battery power), so it is important to extend the lifetime of these devices by minimizing energy consumption. (3) Routing—The routing of data can directly impact the reliability, fault tolerance, and scalability of a HCWSN, and also the energy required by the system for communication. Furthermore, the ability to support multiple receivers and multicast technology is also important, especially in scenarios where a single query is issued (e.g., by a doctor or nurse) to a group of subscribed receivers (e.g., patients). (4) Node Mobility—Both patients and caregivers have the ability to move around and thus exhibit mobility, requiring that the implemented communication layer protocol adapt rapidly to broken routes and fluctuating transmission link quality, and (5) timeliness—On-time delivery of transmitted information is critical, especially in emergency situations.

Many of the aforementioned HCWSN requirements (e.g., reliability, routing, node mobility, *etc.*) have not yet been adequately addressed in a holistic manner by the sensor network community, thus leaving a gap between the existing WSN technologies and the specific requirements. For example, some research has directly addressed WSN reliability concerns by performing an evaluation of efficient and reliable protocols for fixed-power sensor networks [12]. However, the evaluated protocols are not designed to support multicast, mobility, or interference-mitigation. In [13], the TinyOS [14] version of Adaptive Demand-driven Multicast Routing (ADMR [15]), TinyADMR, is employed as the multi-hop routing protocol for CodeBlue, a prototype medical sensor network platform. The results from the experiments offered the following insights regarding the use of a multicast routing protocol in a real life WSN: (1) multicast routing in CodeBlue helps to mitigate the effects of bandwidth limitations. (2) ADMR deals gracefully with node movement for mobility rates typical of moving patients. (3) Background traffic and multiple paths do not adversely affect the packet latency, and (4) redundant packet transmissions increase the PDR. To further build upon the lessons learned from the extensive experiments executed of TinyADMR in CodeBlue, we extend our survey of routing protocols to feature other routing protocols that have been successfully implemented into a HCWSN prototype. By surveying a multitude of different routing protocols optimized for operation in health care applications, we can work closer towards the ultimate goal of producing an effective, efficient, robust, reliable communication layer for HCWSNs.

Energy efficiency is an underlying fabric to the success of deploying WSNs. A significant proportion of energy consumption can be tied to the medium access control (MAC) layer, where energy consuming functions such as idle listening, overhearing, collisions, protocol overhead, and over emitting take place. Like any other application, health care specific applications require an energy efficient MAC protocol to ensure continuous operation along the lifecycle of a biomedical sensor. In this paper, we explore a number of MAC protocols that may meet the requirements of a HCWSN. Specifically, we focus our literature survey on CSMA-based protocols because they generally do not require slot synchronization and slot assignment, resulting in very good scalability.

In addition to energy efficiency, reliability is also paramount to the success of HCWSNs. While redundant transmissions in the form of retransmissions have long been used as a technique for improving reliability, network coding has recently emerged as a viable technology for WSNs. The use of network coding for improving reliability first originated in wired networks. With its success, network coding has been implemented in a number of multicast routing protocols and evaluated in mobile ad hoc networks (MANETs) with good results. Specifically, network coding has been shown to significantly reduce network overhead while also improving PDRs. Reliable transport protocols are also studied as a solution to attaining reliability because they aim to reinforce successful packet delivery in a hop by hop fashion or end to end fashion therefore facilitating the functional operation of reliability-driven applications. Therefore, we review reliable transport layer protocols and a network coding based protocol from the literature for possible integration into a HCWSN.

Recently, Ko *et al.* [16] have published a literature review paper that highlights a multitude of promising healthcare applications and the challenges they pose for integration into a WSN. These challenges arise due to the scarce resource availability inherent of WSNs, and more importantly the requirement to deliver high quality medical data while also ensuring their security and communication privacy. In [16], the aforementioned challenges are analyzed in detail, including the review of several research projects that have been conducted in an effort to overcome these challenges. This paper differs fundamentally from [16] in that it reviews existing techniques that have been proposed in the literature for HCWSNs with the goal of identifying the gaps between their capability and desired HCWSN performance, and then motivate further research to close the gaps. The techniques that were reviewed in this paper span multiple layers including data link (MAC), network (routing), and transport (end to end reliability). The treatment presented in [16] does not encompass multiple layers.

The organization of this paper is as follows: Section 2 provides a survey of major currently existing HCWSNs. Noting that the MAC and routing protocols strongly influence the reliability, energy efficiency and timely data delivery in HCWSNs, the next two sections are devoted to these protocols. Specifically in Section 3, we review the existing MAC protocols and their suitability in a HCWSN. Section 4 details the characteristics and operation of routing protocols that have been implemented in the HCWSNs reviewed in Section 2. In Section 5, several transport layer protocols and a network coding based protocol are reviewed to determine their ability to achieve and maintain end to end reliable communication in HCWSNs. Section 6 offers a discussion of several open issues that require further research. The conclusions are presented in Section 7.

## 2. Survey of Existing HCWSNs

This section provides a comprehensive literature survey of some previously proposed HCWSNs. Specifically, we survey the CodeBlue [13], MEDiSN [19], and MASN [20] HCWSNs because they appear as the most cited projects in the literature, and with sufficient body of knowledge on the five key system architecture requirements that form the central theme of this paper.

**Figure 1.**
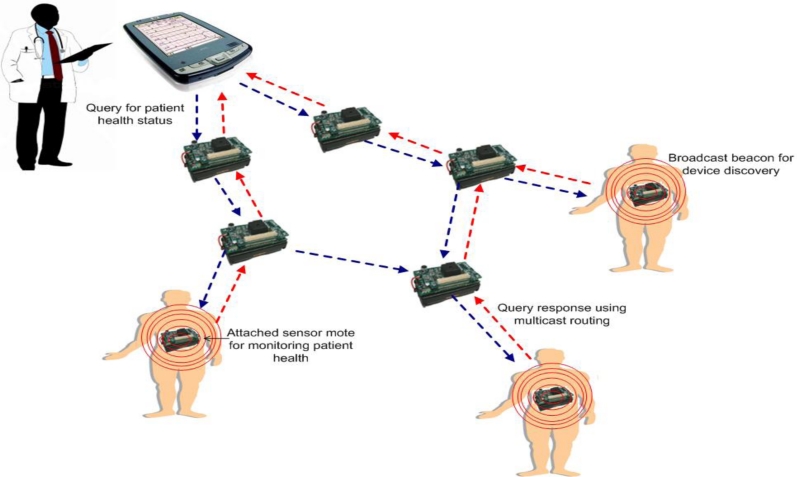
Overview of CodeBlue architecture and operation.

*CodeBlue* [13]: CodeBlue is a prototype health care wireless sensor network that defines an architecture for hardware and a framework for software. The hardware architecture design allows for the integration of a pulse oximeter, electrocardiograph (ECG), and motion analysis sensor board onto the MicaZ [17] and Telos [18] motes. The software framework provides protocols for device discovery, a publish and subscribe routing layer, and a simple query interface that allows caregivers to request data from groups of patients [13]. Figure 1 provides an illustration of the CodeBlue architecture and how it operates.

In Figure 1, each patient is equipped with a sensor mote that is used to monitor health status. A simple device discovery protocol is employed so that the motes in the network can discover each other. Specifically, each node periodically publishes its node ID and the sensor types it supports to a specific broadcast channel using a broadcast beacon. End-user devices, such as personal data assistant (PDA) devices operated by medical professionals (e.g., doctors or nurses), issue network wide queries to request information of patients that are monitored by a group of sensor motes that possess specific biomedical sensing capabilities. In order to facilitate a query and response communication process, the TinyADMR multicast routing protocol is used to establish multicast routes between the data publishing sensors and the end-user devices that subscribe interest to that data. The main purpose of TinyADMR is to deliver queries and responses under the effect of node mobility, multiple simultaneous paths, and an error-prone communication channel. For the querying mechanism, an interface is designed to allow a receiving device to request data from specific biomedical sensors based on their physical node address, sensor type, and whether or not it meets the requirements of a specified filter. CodeBlue is also equipped with a RF-based localization system that is used to track the location of patients and caregivers, a capability that is especially valuable in large hospital settings.

**Figure 2.**
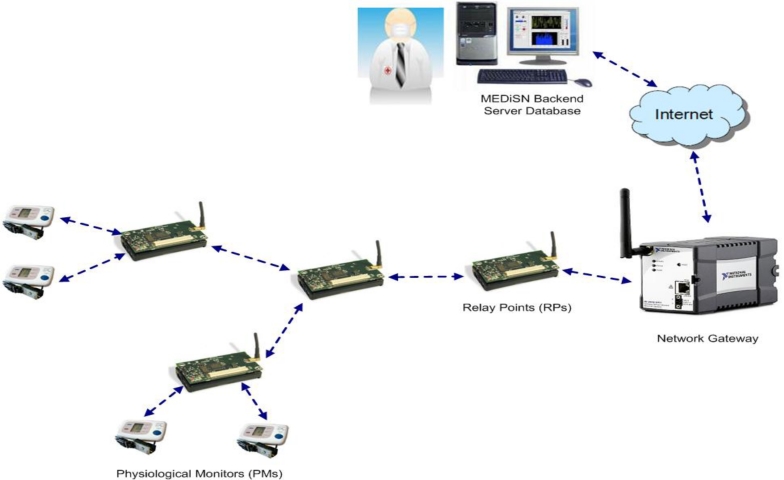
Overview of MEDiSN architecture and operation.

*MEDiSN* [19]: The Medical Emergency Detection in Sensor Networks (MEDiSN) project utilizes a wireless sensor network composed of a network gateway, physiological monitors (PMs), and relay points (RPs), to monitor the health and transmit physiological data of patients. Figure 2 provides an illustrative overview of the MEDiSN architecture and how the various components (e.g., PMs, RPs, *etc.*) operate. The PMs are sensor devices which collect, encrypt and sign patients’ physiological data (e.g., blood oxygen level, pulse, ECG, *etc.*) before transmitting them to a network of relay points that eventually forwards the data to the network gateway. The RPs self organize into a routing tree that facilitates the reliable delivery of periodic data and alerts from the PM to the network gateway, and also from the network gateway to individual PMs. The data received by the network gateway is stored persistently at a backend server, where clients can use a graphical user interface (GUI) to access the data through different queries. Unlike CodeBlue in which the PMs generate and forward data, only the RPs in MEDiSN are responsible for relaying data (*i.e*., PMs in MEDiSN only generate data and are not involved with data forwarding). As a result, the RPs can use hop-by-hop retransmissions to assure the reliable delivery of bidirectional data traffic that is prone to packet collision and corruption. The designations of RPs as the sole nodes with forwarding capabilities allow PMs to duty cycle their radios and reduce their energy consumption. On the other hand, RPs cannot duty cycle because they are actively forwarding packets. However, due to the static nature of the RPs, they can utilize the regular power supply from the hospital or use batteries in scenarios with no infrastructure. Another feature that MEDiSN offers is an over-the-air management interface that remotely controls individual PMs using downstream messaging.

**Figure 3.**
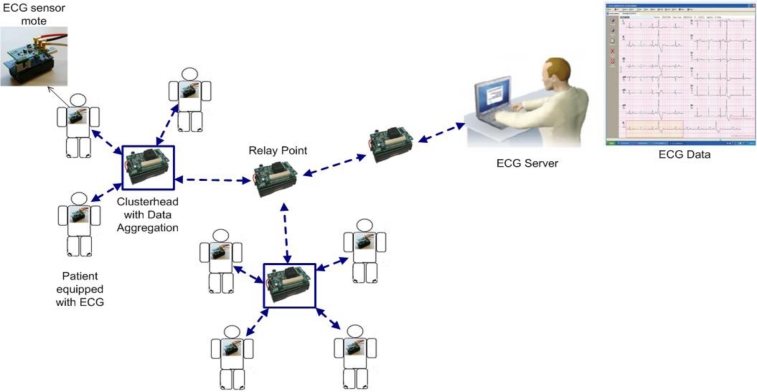
Overview of MASN architecture and operation.

*MASN* [20]: the robust Medical *Ad hoc* Sensor Network (MASN) is a practical hardware and software platform designed to perform real-time collection of health care data. MASN adopts a reliable cluster-based communication scheme as its routing protocol for transmitting data. The protocol groups wireless sensor nodes in clusters to detect signals for the purpose of prolonging the lifetime of MASN, load balancing, and scalability. The clustering scheme reliably relays collected ECG data to the ECG server (sink) in the form of aggregated packets. As a result, it is able to provide fast and accurate event detection and reliability control capabilities to the area where the event is occurring because the overhead, latency, and packet loss are reduced. Figure 3 provides an overview of the MASN architecture and the operation of the different components. As previously mentioned, groups of patients equipped with ECG sensors are organized into clusters. In these clusters, a clusterhead is elected and used as an aggregation point to relay data to the ECG server. After the data is collected, wavelet-based ECG feature extraction and classification techniques are applied to the patient data and characteristic points of interest are extracted. The main benefit of the ECG data mining mechanism is that it provides meaningful information for the diagnosis of possible cardiovascular diseases and also automates the extended recordings of ECG signals, instead of using human processing that is very time consuming and may lead to human errors. To secure the wireless transmission of vital patient data, MASN also employs a low overhead and low complexity encryption and decryption security scheme. The security scheme periodically issues session keys (SK) to cluster heads for secure transmission of data between clusters. Therefore, the security scheme must secure against possible gateway attacks (SKs are issued by the gateway), SK attacks among cluster heads, patient ECG data corruption, and man-in-the-middle attacks [20]. The security scheme successfully defends against these attacks, but does so by adding up to 20% overhead to data packet transmissions [20]. MASN also features a patient location tracking system that is an enhanced version of the CodeBlue MoteTrack [13] algorithm. Using only radio signal information, MoteTrack can determine the location of a patient with an accuracy of 1 meter. The enhanced version of MoteTrack does not use GPS. Rather, the RF chip on each MASN sensor node broadcasts beacon messages at a range of different transmission power levels that the MoteTrack algorithm uses to perform location estimation of patients’ positions.

This section is concluded with an assessment of how well the system characteristics of CodeBlue, MEDiSN, and MASN meet the specific needs of HCWSNs as outlined in Section 1. Table 1 provides a summary of the HCWSN characteristics used to meet the specific needs. Clearly, the performance achieved and therein stated is dependent on each system’s specific operational environment and its particular configuration. As such, Table 1 just summarizes the capabilities of the reviewed systems under their respective operational environments, as reported by their proponents. In terms of reliability, CodeBlue delivers approximately 83% of data packets when the path length between a sender and the receiver is varied between 1 and 6 hops. In MEDiSN, 98% of data packets were delivered when tests were conducted in a real hospital deployment, and the PDR of MASN when there are up to 100 sensors in the network exceeds 90%. Falcon *et al.* [21] states that a reliable HCWSN should have a PDR of at least 90%. Therefore, MEDiSN and MASN meet the reliability requirements while CodeBlue requires additional mechanisms to possibly improve its reliability. Descriptions for energy efficiency were provided only for MEDiSN and MASN. With the use of adaptive duty cycling, the PMs in MEDiSN have a lifetime of approximately 4.62 days, which is a promising result. In MASN, it is shown that most of the sensor battery is consumed in radio communication (65%), which is not energy efficient. This can be largely attributed to the networking protocol design, and some improvements needs to be made to make it more efficient. All three HCWSNs utilize different routing mechanisms. CodeBlue uses the ADMR protocol for multicast routing, MEDiSN optimizes many-to-one and one-to-one communication without explicitly using multicast and MASN uses a cluster-based communication protocol to group sensor nodes. All three systems effectively communicate data from multiple sensors to a sink, so the different routing protocols are acceptable although only CodeBlue uses multicast. The support of node mobility is another key requirement for HCWSNs. CodeBlue deals gracefully with node movement with mobility rates that are typical of patients. MEDiSN succeeded in consistently monitoring patients when they were mobile in a real hospital deployment. Unfortunately, MASN cannot achieve real-time data collection if the patients exhibit mobility because the latency increases substantially as the mobility speed increases. Lastly, we assess the HCWSNs in terms of their ability to deliver packets in a timely manner (<5 s [21]). The end-to-end latency in CodeBlue for paths that spanned up to 7 hops was no more than 200ms. The end-to-end latency in MEDiSN never exceeded 2 s, and the time to reach the sink from the time an event is detected by the sensor node is approximately 1 s for MASN.

## 3. MAC Protocols for HCWSNs

The provision of quality of service (QoS), measured in terms of reliability, latency and energy efficiency, is tightly coupled with the selection of a suitable MAC protocol for the HCWSN. To ensure the longevity of the network and to attain an acceptable end-to-end packet delay, a MAC protocol must strike a balance in the tradeoff that exists between energy efficiency and end-to-end packet delay. For health care applications, it is also important that a MAC protocol account for the mobility that patients may exhibit while under care in their treatment environment. Therefore, when choosing a MAC protocol for a HCWSN, it must be able to adapt to the mobility of nodes that can subsequently result in the change of network size, node density, and network topology. CSMA based MAC protocols aptly adjust to these changing conditions and, thus, are selected for review in this paper. To this end, we briefly review several CSMA-based protocols that also display energy saving and low latency characteristics, and discuss their respective advantages and disadvantages as they relate to their application in a HCWSN.

*S-MAC* [22]: In Sensor-MAC (S-MAC), neighboring nodes form virtual clusters and utilizes local synchronization to set up common sleep schedules. One key feature of S-MAC is its use of message-passing, where a long message is divided into frames and sent sequentially when a node seizes the channel for transmission. Using this technique yields energy savings because less communication overhead is incurred, but at the cost of increased unfairness in the sharing of the medium. The main advantages of S-MAC are that it reduces the energy waste caused by idle listening and also prevents time synchronization overhead with sleep schedule announcements. On the other hand, the sleep and listen periods are predefined and constant, and thus the efficiency of S-MAC is decreased under variable traffic loads. In general, WSNs for health care do not generate very large amounts of data at a time [13,19]. Most of the data is either event-driven or generated periodically in small amounts [13,19]. Therefore, it is possible that S-MAC would not see significant performance degradation in a HCWSN.

*DS-MAC* [23]: Timeliness is a key objective to the success of WSNs for health applications. In Dynamic Sensor-MAC (DS-MAC [23]), a dynamic duty cycle feature is implemented in S-MAC with the goal of decreasing the latency for delay-sensitive applications. Initially, all nodes start with the same duty cycle. During the SYNC period, each node transmits a packet that holds the schedule table which contains the sleep-wakeup cycle of all its neighbors. The header of the packet maintains a one-hop latency value that measures the time difference between when a packet is queued and when the packet is subsequently delivered to the one-hop neighbor of the transmitting node. If a receiver node receives the packet and determines that its latency value exceeds a specified threshold, it doubles its duty cycle by shortening its sleep period length, without modifying its listening period. The main benefit of the dynamic duty cycle adjustment scheme is that it results in lower latency and better scalability than S-MAC, while also delivering more efficient power consumption per packet.

*MD-SMAC* [24]: The Mobility-Aware Dynamic S-MAC (MD-SMAC) protocol is a combination of the mobile version of S-MAC (MS-MAC [25]) and the previously described DS-MAC [23]. The main objective of MD-SMAC is to provide mechanisms to satisfy the constraints posed by delay sensitive applications while also handling mobility conditions in an energy efficient manner. Hameed *et al.* [24] propose modifications to both DS-MAC [23] and MS-MAC to achieve the aforementioned objective. For the DS-MAC [23] component of MD-SMAC, the duty cycle of a node is reverted back to its original duty cycle (*i.e*., duty cycle upon power up) when the energy level threshold *T_E_* has been exceeded. The purpose of this modification is to give priority to extending the lifetime (*i.e*., conserve the remaining energy) of the node by using a small duty cycle, at the cost of increased latency. In MS-MAC, mobile nodes moving from an old cluster to a new cluster maintain both the schedules for the old cluster and that for the new cluster, which is an inefficient use of energy. In MD-SMAC, a modification is made so that the old schedule is removed when a new schedule has been received in the new cluster. Another modification made to MS-MAC is the adaptive change of the neighbor discovery frequency. Normally, when a mobile node moves from one cluster to another, its neighbor discovery frequency increases from once per 5 min (default frequency) to once per 30 s. However, the neighbor discovery frequency never reverts back to the default frequency. In MD-SMAC, a mobile node that has already had its neighbor discovery frequency increased waits to receive its new schedule from the new cluster before reverting back to the default frequency. The collective modifications made to MS-MAC and DS-MAC [23] allows MD-SMAC to possess mobility-aware, delay-sensitive, and energy-efficient characteristics, thus making it strong a candidate for consideration in HCWSNs.

*DS-MAC* [26]: Patients admitted to health care institutions typically have illnesses or injuries of different severity. Therefore, the manner in which traffic is characterized for medical updates from patient to doctor must garner special consideration. The Differential Service Medium Access Control (DS-MAC [26]) protocol accounts for the different traffic types by contextualizing them as normal (Class 2—routine data), warning (Class 1—high priority), and emergency traffic (Class 0—highest priority). This allows DS-MAC [26] to enable prioritized channel provisioning for differentiated service based on the class type. DS-MAC [26] extends the 802.11e QoS MAC protocol by utilizing a preemptive service scheduling algorithm to realize class-based channel access. Each node in the WSN maintains a separate queue for each traffic type. A node contends for channel access with each information frame that it wishes to send. When channel access has been acquired, all packets in the information frame are transmitted successively, unless the service gets interrupted by a higher class traffic type within the same node. By rule, routine data traffic can only be transmitted if both the warning and emergency traffic queues are empty. Otherwise, emergency traffic is always given immediate access for channel service while warning traffic always maintains precedence over routine data traffic. Although this classification of traffic may result in a starvation scenario for routine data, it is still imperative that data concerning medical emergencies are addressed first, because lives may be at stake.

The MAC protocols reviewed in this section offer advantages and disadvantages toward their use in a HCWSN. Table 2 provides a summary of what mechanisms the reviewed MAC protocols utilize in regards to addressing energy efficiency, timeliness, and robustness to mobility. Overall, the MD-SMAC protocol strikes a good balance between energy efficiency and end-to-end packet delay while also handling mobile scenarios. The performance evaluation of MD-SMAC conducted in [24] has shown that it consistently outperforms S-MAC and DS-MAC [23] in terms of disconnection duration, average end-to-end delay, and average energy consumption. In scenarios that evaluated the amount of time a mobile node has been disconnected from the network, both S-MAC and DS-MAC [23] performed poorly because they do not have specific mechanisms to deal with mobility. Although not all nodes in a health care setting will be mobile, it is quite evident that mobility must still be supported. Therefore, future work towards enhancing HCWSN performance via advanced MAC protocols should account for the effect of mobility. In terms of energy consumption, MD-SMAC still manages to outperform S-MAC and DS-MAC [23] even though it employs mechanisms for mobility-handling and delay-handling. The superior performance is due mainly to the modifications made to S-MAC and DS-MAC [23] described above. Yuan *et al.* [26] do not provide any simulation results to evaluate DS-MAC [26]. However, the key detail to extract from DS-MAC [26] is the use of traffic type characterization and prioritization. For health care applications used in environments such as hospitals, it is very important to differentiate data under life threatening conditions from data under stable normal operating conditions. Therefore, traffic type characterization should be considered for MAC protocols designed for a HCWSN.

## 4. Routing Protocols for HCWSNs

In the literature, only a few routing protocols have been defined for HCWSNs. In this section, we describe the routing protocols designed and employed in MASN, CodeBlue, and MEDiSN.

*RMCP* [20]: As part of the MASN platform, Hu *et al.* also proposed a reliable cluster-based communication scheme which is termed Reliable MASN Communication Protocol (RMCP) [20]. RMCP groups wireless sensor nodes into clusters to detect signals for the goal of prolonging the MASN lifetime, load balancing, and scalability. The authors state that RMCP differs from other cluster-based protocols that appear in the literature (e.g., LEACH [27] and HEED [28]), because it takes into consideration the energy level determination of sensor nodes, event-triggered and energy-aware cluster formation, and dynamic adaptation of reliability based on the cluster member density and event proximity. RMCP is a cluster based, energy-aware ECG collection scheme where the ECG data are reliably relayed to a sink node in the form of aggregated data packets. However, unlike many sensor network applications, health care monitoring can only make use of traditional in-network aggregation techniques in certain scenarios, for the collection of a specific type of data. In [20], the authors suggested that ECG data can be appropriately aggregated without compromising the integrity and privacy of the data. The simulation results show that the clustering technique used by RMCP can enable high enough throughput for the good observation of a patient’s health condition. Furthermore, in the scenario where 150 sensors are in the network, MASN can maintain a PDR greater than 80%, making its deployment possibly suitable for a large nursing home. In terms of supporting the mobility pattern and speed of patients and doctors/nurses who move around in a health care environment, MASN is far from ideal as a real-time monitoring system due to excessive wireless transmission delay. For example, when the speed of a node is set to 3.6 kilometers per hour, the end-to-end delay exceeds 5 s [20]. The authors have not offered a solution to address this issue. Furthermore, the simulation results have shown increased reduction in reliability as the source data rate, number of nodes, and mobility are increased, without even considering any effects of interference or fading. In a real life deployment, the performance offered by MASN would be deemed unsuitable.

*TinyADMR* [29]: The developers of CodeBlue use the TinyADMR routing protocol to meet their defined requirements of delivering data reliably and efficiently to multiple receivers. In the route discovery phase of TinyADMR, each node builds a node table that contains an entry for the publisher ID, previous hop node, and estimated cost for the best path from the publisher to the subscriber. To keep the entries in the node table updated, each publisher periodically floods a broadcast ADMR message that is propagated along all intermediate nodes. Each intermediate node that receives the ADMR message first consults its node table. If the estimated path cost from the publisher to the current node is lower than the node table entry, the new previous hop and path cost fields are updated accordingly. The path costs are estimated based on an empirical model that maps the mote’s CC2420 radio’s Link Quality Indicator (LQI) to an estimated link delivery ratio (LDR). For a particular path composed of several links, the PDR is computed as the product of each link’s LDR. The PDR is carried in the header of each ADMR message and updated at each subsequent hop. When a subscriber wishes to receive data from a certain publisher, it sends a *route reply* message along the reverse path from itself to the publishing devices, using the previous hop information in the node table. An intermediate node receiving the route reply message is assigned as a forwarding node and subsequently rebroadcasts it to its neighbors. The performance evaluation of CodeBlue aimed at exploring the effect of increased data rates on achieved throughput. The results showed that TinyADMR achieved a PDR greater than 80% when the data rate was less than 20 packets per second for a network transmission that spanned 2 to 4 hops [29]. For the mobility experiments, ADMR showed robustness to node movement for typical mobility rates and did not suffer a significant drop in PDR. In experiments to measure the packet latency for transmissions across multiple hops, the end-to-end message delay was less than 200 ms in all scenarios. Considering that the latency was measured for scenarios with up to 7 hops, this result is very impressive.

*Modified CTP* [19]: MEDiSN adopts a modified version of the Collection Tree Protocol (CTP) [30] to forward messages through the routing tree structure. CTP’s design is composed of a routing engine and a forwarding engine. The routing engine is used to discover and maintain reliable multi-hop paths between the source and destination nodes. The forwarding engine controls and monitors all transmissions between the RPs and PMs. Specifically, it determines the maximum number of retransmissions required to make the transmission of a packet successful. Results from the literature have indicated that the routing engine of CTP performs well in clinical environments [31]. However, CTP’s forwarding engine incurs high packet loss when the network traffic load is high because CTP allows a high number of retransmissions, which can eventually congest a RP’s packet queue. As a result, Ko *et al.* [19] modify CTP’s forwarding engine to implement an algorithm that dynamically adjusts the maximum number of retransmissions as a function of the RP’s queue size. When the queue size of a RP is above a certain threshold, the number of permissible retransmissions is reduced by half. If the queue size is below a certain threshold and the maximum number of retransmissions has been issued, the maximum number of retransmissions is increased by one. Another modification made to CTP’s forwarding engine is the dynamic adjustment of the inter-packet interval (IPI) that CTP uses. By default, a sender in CTP waits in between 16 ms and 31 ms to send data, which inevitably causes congestion in MEDiSN when high-rate data is transmitted. To counter this problem, the IPI is calculated as an exponentially weighted moving average of all the previous times that a node waited before it successfully transmitted a packet. Unlike RPs, PMs do not use CTP because doing so would force PMs to act as relay nodes and possibly cause expensive routing tree reconfigurations. Instead, patient information packets (PIP) are created at RPs and periodically propagated to the gateway. The PIP contains a list of all PMs that have a connection to a particular RP. When the gateway receives the PIP, it can select the appropriate routing path for transmitting data to the targeted PM. MEDiSN was evaluated in realistic hospital environments and the results showed that MEDiSN succeeded in consistently monitoring the patients even when they were mobile. For example, when MEDiSN was deployed at the University of Maryland Shock Trauma Center, the routing mechanism allowed for a PDR of 98.3% [19]. Furthermore, in the Johns Hopkins Hospital Emergency Room, data was transmitted with a PDR of 95.4% [19].

In this section we have reviewed the characteristics and operation of the RMCP, TinyADMR, and modified CTP routing protocols as they pertain to their respective HCWSNs. A summary of the techniques used by the reviewed routing protocols to address reliability, scalability, latency, and robustness to mobility is provided in Table 3. Of the three routing protocols, RMCP’s performance decreases drastically as the data rate of the source and mobility of the nodes in the network increase. On a positive note, RMCP introduces network aggregation techniques that can be used to support a large number of devices and thus improve scalability. Both TinyADMR and modified CTP have already been integrated into HCWSNs and tested in realistic environments. TinyADMR was evaluated in CodeBlue as part of a 30 node sensor network test bed. The performance results were promising in experiments that dealt with scalability, latency, and mobility. However, the lack of reliable communication has been identified as a gap in the implementation of CodeBlue [13]. TinyADMR is an unreliable multicast routing protocol which allows the system to scale many patient sensor and receiving devices. One technique that can be investigated to improve reliability is redundant transmissions. Preliminary results from Shnayder *et al.* [13] have shown that retransmitting packets increase the PDR, but at the cost of increased bandwidth and end-to-end delay, plus a reduction in energy efficiency. Therefore, optimized techniques for redundant transmission should be further researched. The performance of CodeBlue has been compared with MEDiSN [19] and results show that MEDiSN outperforms CodeBlue both in terms of PDR for the same number of active sensors and also in terms of the maximum number of supported sensors. The authors of MEDiSN provide two lessons learned from their research that can improve the performance of a routing protocol employed in a HCWSN: (1) Congestive losses and end-to-end transmission delays can be minimized by dynamically adjusting the maximum number of retransmissions that relay nodes issue and by computing the optimal IPI, and (2) The division of functionality between acquiring (PM) and relaying data (RP) enables PMs to achieve consistent and predictable behavior. As a result, a well engineered wireless backbone with strategically deployed relay points can improve PDRs up to threefold [19].

## 5. End-to-End Reliable Transmission Mechanisms for HCWSNs

There are two common approaches suggested in the literature to improve end-to-end reliability in HCWSNs. One approach explores the use of reliable transport protocols [32] and the other explores the use of redundant transmissions and coding techniques [33] to allow data to be reconstructed at the receiver. In the following subsections, we explore both options in further detail.

### 5.1. Reliable Transport Layer Protocols

Due to the scarce bandwidth, energy constraints, and unpredictable characteristics of the wireless medium in WSNs, end-to-end reliable transport protocols like the transmission control protocol (TCP) are not suitable [32]. Nevertheless, several reliable transport protocols have been proposed for WSNs. The protocols can be categorized in terms of the direction (*i.e*., upstream or downstream) in which reliability can be obtained. In the context of health care, downstream is used for queries from the server (e.g., doctors) to the sensors (e.g., patients) and upstream is used for events detected at the sensors and transmitted to the server. It is also important that the reliable transport protocol support multicast transmissions for the delivery of data from a single server to several patients. For this reason, we focus our review on multicast-capable protocols.

*PSFQ* [34]: Pump Slow Fetch Quickly (PSFQ) is a downstream multicast and broadcast reliable transport protocol for WSNs that performs hop by hop error recovery. PSFQ offers four key features: (1) it is independent of the routing layer. (2) Conserves power by both using hop by hop error recovery and NACKs to prevent NACK implosion problems. (3) Adopts multimodal operation to adaptively account for low and high error rates, and (4) the aggregation of errors, minimum retransmission and retransmission requests make it scalable and efficient. As its name implies, PSFQ distributes data slowly (pump slow) and recovers from errors quickly (fetch quick). In the pump process, the source node periodically broadcasts a data packet every *T_min_*. When the packet is received by a node, it forwards the packet at any time between *T_min_* and *T_max_*, delaying the transmission by *T_min_* to allow for the fetch quick operation. When this transmission occurs over multicast paths that have several hops, the delay can be high. Each transmitted packet from the source node contains a unique sequence number. When a receiver node receives a packet with a sequence number that is out of sequence, the fetch mode is entered. While in fetch mode, the receiver node generates and broadcasts a NACK message (which contains the sequence numbers of the packets that are missing) to its one-hop neighbors every *T_r_* until the missing packets have been recovered or the maximum number of requests have been issued. The neighboring nodes retransmit the missing packet to the requestor if they have the packet stored in their data cache. The simulation results for PSFQ show that the PDR and latency do not degrade but increase, respectively, with increasing hops at high error rates (30%) [34]. Furthermore, when the error rate is less than 70%, the retransmission overhead only increases by a small margin. These results show that PSFQ exhibits high error tolerance and is able to scale to a large number of hops without much performance degradation. In health care applications where error rates can be high due to obstructions, interference, and mobility, it is necessary for the reliable transport protocols to be robust to errors.

*GARUDA* [35]: GARUDA addresses the problem of reliable downstream point-to-multipoint data delivery in WSNs using a scalable framework that leverages the characteristics of a WSN while achieving reliability in an efficient manner. There are three main elements that define the framework of GARUDA’s design: (1) single/first packet delivery. (2) Instantaneous core construction, and (3) two phase loss recovery. GARUDA ensures the reliable delivery of the first packet of messages of any size using a pulsing based approach, and thus is not susceptible to the “all packets lost” problem [35] that most NACK based schemes endure. To ensure the delivery of the first packet, a process called Wait-for-First-Packet (WFP) pulse transmission takes place. In this process, the WFP pulse, which is a short period signal that is twice the regular transmission power, is propagated to all nodes in the network to notify them that the first packet is ensuing. Each node continues the WFP pulsing until it has received the first packet. If it does not receive the first packet within a certain time, a NACK is sent to its single hop neighbor to request for retransmission. During the delivery of the first packet, a single packet flood approach is used to dynamically assign a subset of nodes to function as loss recovery servers. These nodes are termed core nodes and their distributed designation represents an approximation of the minimum dominating set (MDS) of the network sub-graph to which reliable message delivery and the recovery of lost packets may be required. When the core nodes have been designated, GARUDA employs a two-stage loss recovery process that first deals with core nodes recovering from lost packets, then non-core nodes recovering from lost packets. The loss recovery process for core nodes is conducted in unison with the default message forwarding process, and all requests and retransmissions for lost packets are performed as unicast transmissions to the nearest upstream core that has the packet stored in its cache. In the loss recovery process for non-core nodes, the non-core nodes wait for an indication from the core nodes that they have received all the packets in a message. When this indication is received, the non-core node first checks its core node table for the core node that has the closest hop distance to it. The non-core node then initiates the recovery process through the nearest code node. With regards to the defined requirements for HCWSNs, GARUDA addresses the need for a reliable transport protocol that supports a multicast paradigm where a sink node can reliably deliver data to a group of sensors belonging to the same multicast group. The performance evaluation shows that GARUDA exhibits significantly lower latencies when compared to other ACK and NACK based transport protocols, and also exhibits significantly better energy consumption efficiency as the number of sent data packets is increased [35]. GARUDA offers amenable features that could well benefit its adoption in a health care based WSN. However, there is no explicit mechanism to account for different mobility, which could result in a degradation of performance.

*ART* [36]: The Asymmetric and Reliable Transport (ART) mechanism was proposed to address bidirectional end-to-end reliability in wireless sensor networks. In WSNs, bidirectional reliability relates to reliably transmitting detected event messages to the sink and reliably transmitting query messages to the sensors. To accomplish this feat, ART utilizes an energy-aware classification algorithm that groups sensors into essential nodes (E-Nodes). The essential nodes are chosen to provide sensing coverage of a designated area. The remaining nodes are called non-essential nodes (N-Nodes). The energy-aware classification algorithm is based on a greedy-weighted algorithm that determines the remaining battery power on each node. This information is used to greatly reduce the probability of a low-power node becoming an essential node. As a result, the available energy in the network can be more balanced and thus the network lifetime can be prolonged. To facilitate reliable event and query transfer, ART uses asymmetric lightweight ACK and NACK mechanisms, respectively, between the sink and essential nodes. The queries sent by the sink to the sensors are sequentially ordered. Therefore, if a sensor detects a gap in the received queries or if the queries are received out of order, it is assumed a loss has occurred. To recover the lost message, the sensor sends a NACK to the sink node. For reliable event transfer, the E-Nodes only require an acknowledgement from the sink node for event messages that have an event notification bit set. These event messages are typically the first messages that are sent out by an E-Node sensor when a new event is detected (e.g., a new value is sensed). When the sink node receives the event message with the event notification bit set, it responds with an ACK to the E-Node. If the ACK is not received by the E-Node within a certain timeout period, the distributed congestion control algorithm is used to send *congestion alarm* messages to non-essential nodes. The congestion alarm messages provide instruction that the traffic of N-Nodes should be reduced (*i.e*., temporarily stop the sending of sensed data). When an ACK is finally received by the E-Node, a *congestion safe* message is broadcast to announce that normal operation of the network can resume. The simulation results have shown that under a 100% reliable PDR objective, the traffic load in the network can be drastically reduced [36]. Furthermore, ART performs better than other message-level reliability schemes in terms of latency and packet loss [36].

The performance evaluations for PSFQ, GARUDA, and ART have used different experiments to measure the effectiveness of the reliable transport protocols [34–36]. Table 4 provides a summary of techniques used the aforementioned protocols to address reliability, energy efficiency, latency, and scalability. In PSFQ, PDR and latency are measured as a function of the network size (number of hops). PSFQ achieves 100% delivery up to ten hops away from the source node when the error rate is 50%, and more than 90% delivery for paths 13 or more hops away [34]. As the number of hops across the network increases from 1 to 5, the average latency increases linearly from 175 ms to approximately 325 ms [34]. In the experiments for GARUDA, latency and energy consumption are measured as a function of node density (*i.e*., scalability experiment). For multiple-packet delivery, the latency and energy consumption scale well with an increase in node density. For example, as the node density increases from 200 to 800, the latency only increases from 5 s to 13 s and the energy consumption increases from 5 J to 9 J [35]. In ART, simulation results are provided for network lifetime under light and heavy traffic conditions with varying sensor node update intervals. For a network of 100 nodes that periodically update every 30 s, the network lifetime is approximately 225 s, irrespective of the traffic level [35]. Obviously, for a prototype HCWSN, this value is not favorable because the first battery operated sensor will have to be replaced in less than 4 min. However, simulating ART under different conditions may reveal an extended life time. The simulation results for ART also show that under heavy traffic load conditions where the node density is 200, the end-to-end delay remains less than 5 s and the PDR is greater than 90% [36]. The solid performance of ART is due to the advantage gained by having designated E-Nodes deal with retransmissions that reduces the amount of data in the network.

In HCWSNs, it is imperative to provide reliable data transfer both upstream and downstream. The ability for a doctor to query a patient is equally as important as a patient’s ability to have his or her measurements sent to the doctor or a processing server. Therefore, a reliable transport protocol that features an ability to ensure reliable upstream and downstream data transfer while also supporting multicast communication is most ideal for integration in a HCWSN. While GARUDA and PSFQ support multicast communication, they are limited in the context of supporting HCWSNs because reliability is only provided downstream. On the other hand, ART also supports multicast (and unicast) transmissions while providing reliability for upstream events and downstream queries. The results of the performance evaluation show that the distributed congestion control mechanism effectively mitigates congestion in the network. Furthermore, when the network node density is less than 100, the end-to-end delay and PDR are at an acceptable level (<5 s and >90%, respectively [21]). However, additional work is required to improve the network lifetime of ART.

### 5.2. Network Coding Techniques for Improved Reliability

Efforts from the literature to use network coding as a means to improve reliability in wireless networks are briefly reviewed in this subsection. Application of the network coding concept to WSN is relatively new, hence, not much work currently exist in the literature. A network coding based protocol from the literature that is specifically designed for WSNs is AdapCode [33]. AdapCode is a reliable data dissemination protocol that uses adaptive network coding to reduce broadcast traffic in the process of code distribution updates in WSNs. Specifically, AdapCode achieves high reliability by adaptively changing the network coding scheme according to perceived link quality. The analysis in [33] shows that the capability for and the process of network coding for a WSN protocol is not too resource taxing or memory draining for WSN nodes because network coding only imposes a 3 byte header overhead, linearly independent packets are easily retrieved, and Gaussian elimination only requires 1 kilobyte of memory. Unfortunately, AdapCode may not be fully suitable for the health care applications considered in this paper because it does not support multicast and also does not adapt to network dynamics induced by mobility. Nevertheless, AdapCode has shown that the application of network coding in WSNs can be beneficial towards improved performance in HCWSNs. Thus, future integration of network coding into a multicast routing protocol should be explored.

## 6. Discussion

In this section, we identify and discuss specific areas for further research to close the gaps that have been identified in this paper. Researchers have taken a major step towards enhancing the performance of HCWSNs in terms of reliability, mobility awareness, and timeliness, with the overall goal of minimizing the persisting gaps. However, future work is required to specifically analyze the energy efficiency of HCWSNs, without applying the common assumption that a certain number of sensor nodes in HCWSNs have access to external power supplies. While this assumption normally holds true in environments such as hospitals and nursing homes, the same cannot be said for scenarios where triage is immediately required at disaster sites. Therefore, experiments are required to determine specifically how much energy fully operational battery-driven biomedical sensors consume, and the resulting lifetime duration of the HCWSNs.

MAC protocols are typically employed to ensure energy efficiency and fair access to the transmission medium. However, MAC protocols must also consider the different traffic types that are characteristic of sensor-equipped patients experiencing various degrees of medical distress (e.g., life threatening conditions *vs.* minor illness). In a HCWSN, special consideration must be afforded to data traffic of higher priority. Thus, a MAC protocol scheme where a sensor node is granted access to the wireless medium based on traffic prioritization must be further investigated. It is pertinent that the MAC protocols implementing traffic prioritization achieve the desired performance objectives such as good energy efficiency and low packet transmission latency while evaluated under different operational scenarios such as different scalability and mobility characteristics.

Data aggregation is a common technique utilized by routing protocols for the purpose of reducing transmissions and increasing throughput. However, in most cases, routing protocols that adopt data aggregation schemes are either organized in clusters or hierarchies where a node (e.g., cluster head) is designated as an aggregation point [37]. In HCWSNs that are modeled using a flat network architecture, an aggregation point is typically not designated *a priori*. Therefore, a routing protocol that adopts a scheme to dynamically identify candidate nodes in the network that present an opportunity for in-network aggregation would be beneficial for the following reason: nodes that remain static for long periods of time in the network (e.g., a group of patients recovering from similar intensive operations such as blood transfusions) periodically broadcast unchanged routing information. As such, the routing information from the group of similar acting nodes can be aggregated and propagated as a single transmission.

The benefit of cross-layer design (CLD), applied to conventional WSNs, in terms of QoS support without a loss of energy efficiency has been demonstrated in previous works [38,39]. We conjecture that CLD will also enable HCWSN QoS requirements (e.g., >90% PDR and 5 s latency [21]) for health care applications to be met. In the context of the three protocol layers reviewed in this paper, information must be exchanged between and across the MAC, routing and transport layers. What and how information should be exchanged along with the benefit-to-cost ratio of cross-layer design in the context of HCWSNs remain an open issue for further investigations.

The use of network coding to enhance reliability in HCWSNs has not been investigated, to our knowledge. Assessing the effectiveness of network coding in a HCWSN requires further detailed investigation. Minimum cost multicast algorithms [40,41] that adopt network coding have been proposed for wireless networks. Based on this work, future work can be performed to assess the effectiveness of the aforementioned algorithms when adapted to the characteristics of a wireless sensor network. Furthermore, since the concept of network coding increases the amount of consumed energy, it is imperative to investigate whether or not all nodes in the network are required to perform network coding, or if a specific set can perform the network coding, without significant loss in overall performance. Lastly, network coding also has a propensity to increase end-to-end latency. Thus, different techniques for network coding (e.g., configuring the number of buffered packets) should be investigated, to possibly limit latency.

As the design and requirements for a more reliable HCWSN become more complex and diversified, the effort towards ensuring the secure transmission of private and delicate medical data must also persist. CodeBlue and MASN have implemented security schemes to provide security and privacy of data transmission. Specifically, CodeBlue has implemented Elliptic Curve Cryptography (ECC) on its MICA2 motes only using integer arithmetic [13]. Unfortunately, the time to generate an encryption key takes 35 s, which is deemed unsatisfactory [42]. MASN has implemented a trustworthy medical transmission scheme to promote confidentiality and integrity of patient data through the use of crypto-keys. However, the security design is only limited to one-hop ECG data transmission. Ko *et al.* have stated that the integration of security protocols into MEDiSN remains a top priority. While no recent literature appears in which the transmission of data in MEDiSN has been fortified with a relevant security scheme, Huang *et al.* [43] proposed a pervasive, secure access scheme for a hierarchical-based architecture that closely matches that of MEDiSN. The performance results in [43] show that, using a symmetrical key cryptosystem, the transmission of private data can be kept secure while only slightly degrading overall system performance. The following future work must be pursued to enhance the security of CodeBlue, MASN, and MEDiSN: For CodeBlue, a form of cryptography improving the complexity of integer arithmetic should be explored. In the case of MASN, the single hop security design for ECG data transmission must be extended to a multi-hop scheme. Finally, the adoption of the security scheme proposed in [43] into MEDiSN should be pursued as future work.

## 7. Conclusions

The WSN research community has done an admirable job of addressing some of the limitations that currently exist for health care related applications. Proposals have mostly focused on the deployment of tiny wearable medical sensors, while others have developed infrastructures for monitoring individual patients during daily activity, at home, or at a hospital. In this paper, we review the state-of-the-art in wireless sensor network research and highlight the gaps between the existing technologies and the needs of a Health Care Wireless Sensor Network (HCWSN), with special emphasis on reliable communication. A survey of the existing HCWSNs reveals that MEDiSN [19] offers the most suitable and comprehensive system for the facilitation of reliable communication. MEDiSN adopts a multi-tiered architecture that uses a planned wireless backbone network to fortify network throughput and achieve high PDRs. Reliable communication in health care applications also encompasses the delivery of packet with low latency. We reviewed a number of MAC protocols from the literature that struck a good balance between energy efficiency and low latency packet transmission. We found that MD-SMAC was the most suitable for HCWSNs because it uses a combination of dynamic duty cycling and adaptive neighbor discovery frequency to handle mobility scenarios. Furthermore, MD-SMAC is able to achieve energy efficiency while not incurring exorbitantly high delivery latency. We also examined the performance of a number of different routing protocols employed in HCWSNs. Our comparisons showed that the routing protocol in MEDiSN and modified CTP outperforms the routing protocol in CodeBlue and TinyADMR, in terms of PDR for the same number of active sensors and also in terms of the maximum number of supported sensors. To ensure the QoS requirements (>90% PDR and 5 second latency [21]) of health care applications are met, we also investigated various mechanisms for reliable transmission. We concluded that the ART protocol is most adequate for HCWSNs mainly because it supports multicast transmissions and can provide reliability for upstream events and downstream queries. Finally, we consider network coding as an alternative to improving reliability, and the results from the performance evaluation of AdapCode show that network coding can increase PDR while also lowering the level of expended overhead (improved energy efficiency).

## Figures and Tables

**Table 1.** Characteristics of CodeBlue, MEDiSN and MASN HCWSNs.

**Requirements**	**CodeBlue [13]**	**MEDiSN [19]**	**MASN [20]**

Operational Environment	30 Node *Ad Hoc* Sensor Network Test-Bed	Dedicated Wireless Sensor Network in Hospital Deployment with 6 RPs and 8 PMs	Simulation-based *Ad Hoc* Sensor Network
Supported Application	Medical Care and Disaster Response	Emergency Detection	Real-time collection of ECG Data
Reliability Mechanism	None (Unreliable Multicast)	Two-Tier Architecture with Dedicated Wireless Backbone and Optimized Rate Control Protocols	Dynamic Reliability Adaptation Scheme
Scheme for Energy Efficiency	Not Provided	Division of functionality between acquiring (PM) and relaying (RP) data	Energy-aware cluster formation using energy level determination of sensor nodes
Routing Methodology	Multicast	Many-to-one and one-to-one communication	Intra-Cluster and Inter-Cluster Data Relay
Techniques for Mobility Support	Periodic Flooding for Route Discovery	PMs periodically select the best RP to forward their data to	None. Does not support real-time data collection under mobility conditions

**Table 2.** Summary of potential CSMA-based protocols for HCWSN.

**Criteria**	**S-MAC [22]**	**DS-MAC [23]**	**MD-SMAC [24]**	**DS-MAC [26]**

Key Feature	Message-passing	Dynamic duty cycle	Dynamic duty cycle and adaptive neighbor discovery frequency	Prioritized channel provisioning for differentiated service
Energy Efficiency	(+) Reduces energy waste caused by idle listening (–) Poor under variable traffic loads	(+) Automatically adjust duty cycle based on current energy consumption level	(+) Prioritizes energy efficiency by reducing duty cycle when energy level threshold has been exceeded	No discussion presented in [26]
Timeliness (Latency)	(–) Unfairness in the sharing of the medium, leading to extended transmission delays	(+) Autonomous duty cycle adjustment prevents latency from increasing when delay threshold at destination has been met	(–) Aims to extend network lifetime at expense of increased latency	(+) Service preemption and shorter back-off duration allows emergency data to win channel access when competing with normal data
Robustness to Mobility	(–) Designed for stationary scenarios. Nodes are disconnected from network for 10 s every 2 min	(+) Multiple duty cycles and dynamic duty cycling allows for adjustment to different update intervals (–) No evidence to show that the dynamic duty cycle can handle different mobility conditions	(+) Adaptive neighbor discovery frequency keeps the mobile node connected through different mobility speeds	No discussion presented in [26]

**Table 3.** Summary of routing protocols for HCWSN.

**Criteria**	**RMCP [20]**	**TinyADMR [29]**	**Modified CTP [19]**

Reliability (in terms of PDR)	(+) Dynamic adaptation of reliability based on the cluster member density and event proximity	(+) PDR is very high for data rates below 5 packets per second with 10 senders transmitting data over multiple hops	(+) Proposed RP selection scheme allows PMs to connect to a more reliable RP once initial connectivity has been lost
Scalability	(+) Supports a large number of nodes due to event-triggered and energy-aware cluster formation	(+) System can scale to a large number of devices each with a modest data generation rate.	(+) Can support at least five hundred physiological monitoring sensors (PM) depending on the amount of data each PM generates
Timeliness (Latency)	(–) Time required for data packet aggregation severely hinders end-to-end latency	(+) End-to-end message delay less than 200 ms for destinations up to 7 hops away	(+) Delay is minimized using dynamic adjustment of retransmissions and computing optimal inter-packet arrival time at RPs
Robust to Mobility	(–) Cannot achieve real-time data collection if user moves quickly	(+) Deals gracefully with node movement for mobility rates typical of walking or moving patients	(+) The dedicated wireless backbone architecture effectively masks the effects of mobility

**Table 4.** Summary of reliable transport protocols for suitability in HCWSN.

**Criteria**	**PSFQ [34]**	**GARUDA [35]**	**ART [36]**

Reliability (in terms of PDR)	(+) PDR increases with increasing hops at high error rates (30%)	(+) Targets 100% reliability within a sub region, to cover the sensing field, and to a probabilistic subset	(+) Targets 100% query and event reliability using end-to-end ACK/NACK
Energy Efficiency	(+) Conserves power by using hop by hop error recovery	(+) Avoids NACK implosion and high energy consumption by invoking WFP pulses	(+) Balances available energy amongst nodes using energy-aware node classification algorithm
Timeliness (Latency)	(+) Fetch Quickly scheme allows for acceptable latency when error rate is very high (70%) (–) Pump Slowly scheme degrades latency performance when error rate is around 30%	(+) Uses a locally designated server and out-of-sequence forwarding to reduce latency	(+) Reduced delay due to classified E-Nodes minimizing amount of data sent and using event-based reliability to avoid ACK implosion
Scalability	(+) Aggregation of error, minimum retransmissions, and transmissions requests allows network to handle more data packets	(+) Scalable with respect to network size, packet loss rate, reliability semantics, and message characteristics	(+) Effectively manages increased node density since only E-nodes are used to address congestion
